# Combinatorial Approaches to Restore Corticospinal Function after Spinal Cord Injury

**DOI:** 10.1523/ENEURO.0185-25.2025

**Published:** 2025-06-12

**Authors:** Najet Serradj, Edmund Hollis

**Affiliations:** ^1^Burke Neurological Institute, White Plains, New York 10605; ^2^Feil Family Brain and Mind Research Institute, Weill Cornell Medicine, New York 10065

Spinal cord injury (SCI) results in the loss of sensory and motor functions due to the inability of mature central nervous system (CNS) neurons to regenerate. Developing robust neural regrowth strategies will be critical for re-establishing corticospinal motor neuron circuits and restoring control over voluntary movement. However, the complex nature of SCI necessitates a multifaceted approach to address several key barriers to regeneration: enhancing the limited intrinsic growth ability of injured adult neurons, mitigating the growth inhibitory signals of the injured spinal cord, and providing a growth-permissive substrate.

The intrinsic capacity for axons to regenerate declines precipitously in early postnatal development. There are numerous changes in transcriptional control, epigenetic regulation, cell signaling, and metabolism with CNS maturation (Zheng and Tuszynski, 2023). One well defined change is a decline in growth-promoting phosphatidylinositol 3-kinase (PI3K) signaling as phosphatase and tensin homolog (PTEN) dephosphorylates phosphatidylinositol 3,4,5-triphosphate to negatively regulate mammalian target of rapamycin (mTOR; Liu et al., 2010). Aberrant activation of PI3K signaling has been used in multiple studies to enhance CNS axon regeneration (Nieuwenhuis and Eva, 2022).

**Figure 1.**
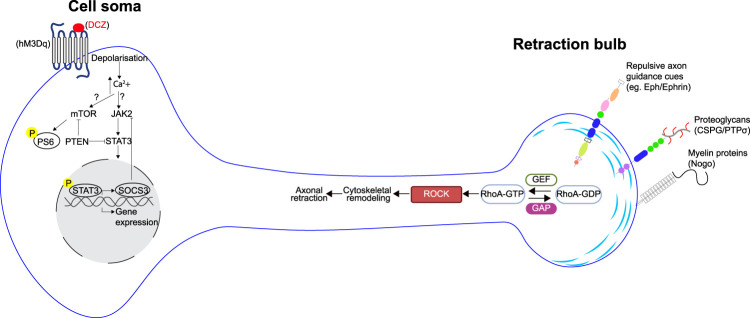
Targeting multiple barriers to axon regeneration. A combinatorial approach was used by Takatani et al. to activate neuronal growth via chemogenetic activation while depleting PTEN and RhoA. In this approach, DREADD (hM3Dq) activation with DCZ drives depolarization and activates mTOR through calcium influx. Similarly, chemogenetic stimulation increases JAK activity, which then activates STAT-mediated transcriptional regulation. In addition, the authors targeted PTEN and RhoA via conditional deletion. PTEN loss leads to hyperactivation of mTOR. After axon injury, RhoA is activated by RhoA-GEFs in response to various extracellular signals. Active GTP RhoA stimulates downstream effector protein ROCK, which drives cytoskeleton remodeling through intracellular signaling cascades leading to axon retraction. DCZ, deschloroclozapine; GAP, GTPase-activating protein; GEF, guanine nucleotide exchange factor; hM3Dq, human Gq-coupled M3 muscarinic receptor; JAK2, janus kinase2; mTOR, mammalian target of rapamycin; PTEN, phosphatase and tensin homolog; PS6, ribosomal protein S6 phosphorylated at s235/s236 residues; ROCK, Rho kinase; SOCS3, suppressor of cytokine signaling3; STAT3, signal transducer and activator of transcription 3.

Numerous neuron extrinsic cues in the injured spinal cord limit axon regeneration (e.g., myelin-associated proteins, chondroitin sulfate proteoglycans, repulsive guidance molecules), several of which converge on Rho-GTPase signaling pathways (Giger et al., 2010). In the CNS, the small GTPase Ras homolog member A (RhoA) is a central mediator of axon retraction after SCI (Fig. 1). RhoA activates the downstream effector Rho-associated kinase (ROCK) which negatively affects the axon structure by modulating cytoskeletal dynamics and tubulin depolymerization, making RhoA inhibition a promising target for reducing axon retraction after SCI.

Recognizing the intrinsic regenerative differences between central and peripheral nervous systems, the grafting of growth-permissive substrates was perhaps the earliest approach to elicit CNS axon regeneration, with pioneering studies by Tello and Cajal, revisited by David and Aguayo with modern tracing techniques. While several substrates and cell types have been found to provide suitable substrates for CNS axon regeneration, recent efforts with embryonic and induced stem cell grafts have been found to not only support corticospinal tract (CST) axon regeneration into grafts but also to serve as bridges with graft axons extending well into host tissues (Zheng and Tuszynski, 2023). Perhaps most intriguing is that neural progenitor cell grafts appear to both provide extrinsic cues to shape CST axon regeneration and regulate the intrinsic growth capacity of injured neurons (Zheng and Tuszynski, 2023). Furthermore, heterogeneous embryonic grafts show regions that exclude corticospinal axons in an anatomically relevant manner, indicating the likely role of the molecular mechanisms that shape spinal cord development in controlling regeneration (Dulin et al., 2018).

In a recent study, Takatani et al. have sought to combine interventions to overcome these barriers (Takatani et al., 2025) by building upon their prior efforts to both enhance intrinsic regeneration through the genetic deletion of *Pten* and to limit the response of corticospinal neurons to extrinsic repulsive cues through deletion of *RhoA* (Nakamura et al., 2021). In the current study, the authors used an intersectional viral approach to selectively drive activity in corticospinal neurons in combination with *RhoA,Pten* double conditional knock-out. By delivering retrogradely transported AAV encoding Flp recombinase to the cervical spinal cord, they expressed the excitatory chemogenetic receptor hM3Dq in a subset of corticospinal neurons in the forelimb sensorimotor cortex using a local, Flp-dependent vector. Daily intraperitoneal injections of the second-generation DREADD ligand deschloroclozapine (DCZ) were then used to elevate baseline levels of activity in injured corticospinal neurons and they evaluated the effects on axon retraction, collateral growth, and grid walking behavior.

Chemogenetic stimulation was used to mimic the growth-promoting effects of cortical electrical stimulation but restricted to an anatomically defined subset of corticospinal neurons. Cortical electrical stimulation has been shown to enhance the intrinsic growth capacity of corticospinal axons by activating Jak/Stat and Ras/ERK signaling pathways, in addition to mTOR (Zareen et al., 2024). Takatani et al. showed that in response to a bilateral, cervical, dorsal column SCI, the combinatorial treatment of chemogenetic activation with *RhoA,Pten* double conditional knock-out reduced CST axon retraction, led to increased density of bouton-like structures in injured axons caudal to the lesion, and supported a modest level of behavioral improvements over *RhoA,Pten* double conditional knock-out alone.

The limited effects of chemogenetic cortical activation in addition to *RhoA,Pten* double conditional knock-out may be a limitation of the method of activation. In a pyramidotomy model of CST axon remodeling in mice, continuous DREADD activation has previously been shown to evoke no discernable effect on either sprouting or CST-dependent behavioral recovery (Wang et al., 2024). In a direct comparison of selective corticospinal neuron activation by either patterned intermittent theta-burst activation by optogenetic stimulation or nonpatterned chemogenetic activation in intact rats, patterned optogenetic stimulation drove changes in connectivity with appropriate CST target neurons, whereas nonpatterned chemogenetic activation drove similar levels of axonal sprouting without appropriate targeting (Yang and Martin, 2023). This disparity could underlie the functional limitation of chemogenetic approaches to mimic cortical electrical stimulation, as neuronal activity may not only need to be stimulated but also be temporally regulated. Optogenetic stimulation directly depolarizes neurons, while chemogenetic techniques act by reducing the threshold for action potential propagation. Similarly, intermittent patterned electrical stimulation results in a greater physiological plasticity than repetitive high-frequency stimulation (Zareen et al., 2024). If mechanisms of Hebbian plasticity underlie the rewiring of injured corticospinal axons, then methods to mimic the appropriate time-locked patterns of corticospinal activity may be required to sufficiently strengthen de novo circuits to effectively support behavioral recovery.

While this study demonstrates some moderate effects on axon plasticity in a mouse model of SCI, it also illustrates several remaining challenges. Mice do not develop cystic cavitation after SCI as other mammalian species do, including humans, so these studies did not include a growth-permissive substrate in the lesion site. Despite the absence of a lesion cavity, this combinatorial treatment supports reduced axon retraction and local plasticity, not axon regeneration through the lesion core. Furthermore, the translational relevance is unclear as manipulation of the oncogenic mTORr pathway has limited translational potential since PTEN deletion leads to hypertrophic cell somata and axons. Additionally, cortical stimulation in humans will likely be limited to noninvasive transcranial magnetic stimulation. Considering the goals of developing axon growth-based therapeutics, it is unlikely that synaptogenesis alone will be sufficient to support recovery. Additional refinement and remyelination of corticospinal circuits will likely be required to improve movement recovery.
